# Supplementary material for: A Facile Strategy for the Preparation of N-Doped TiO2 with Oxygen Vacancy via the Annealing Treatment with Urea
Source: Nanomaterials (Basel). 2024 May 7;14(10):818. doi: 10.3390/nano14100818 (PMC11123904; doi:10.3390/nano14100818)
Supplement: Supplementary file 1 [file nanomaterials-14-00818-s001.zip › nanomaterials-2971610-supplementary.pdf]

nanomaterials

Supporting Materials

A Facile Strategy for the Preparation of N-Doped TiO_2 with Oxygen Vacancy via the Annealing Treatment with Urea

Zhe Zhang [1], Zhenpeng Cui [1,2,*], Yinghao Xu [1], Mohamed Nawfal Ghazzal [3], Christophe Colbeau-Justin [3], Duoqiang Pan [1,2] and Wangsuo Wu [1,2]

[1] School of Nuclear Science and Technology, Lanzhou University, Lanzhou 730000, China
[2] Frontiers Science Center for Rare Isotopes, Lanzhou University, Lanzhou 730000, China
[3] Institute of Physical Chemistry, Paris-Saclay University, 91405 Orsay, France
* Correspondence: cuizp@lzu.edu.cn

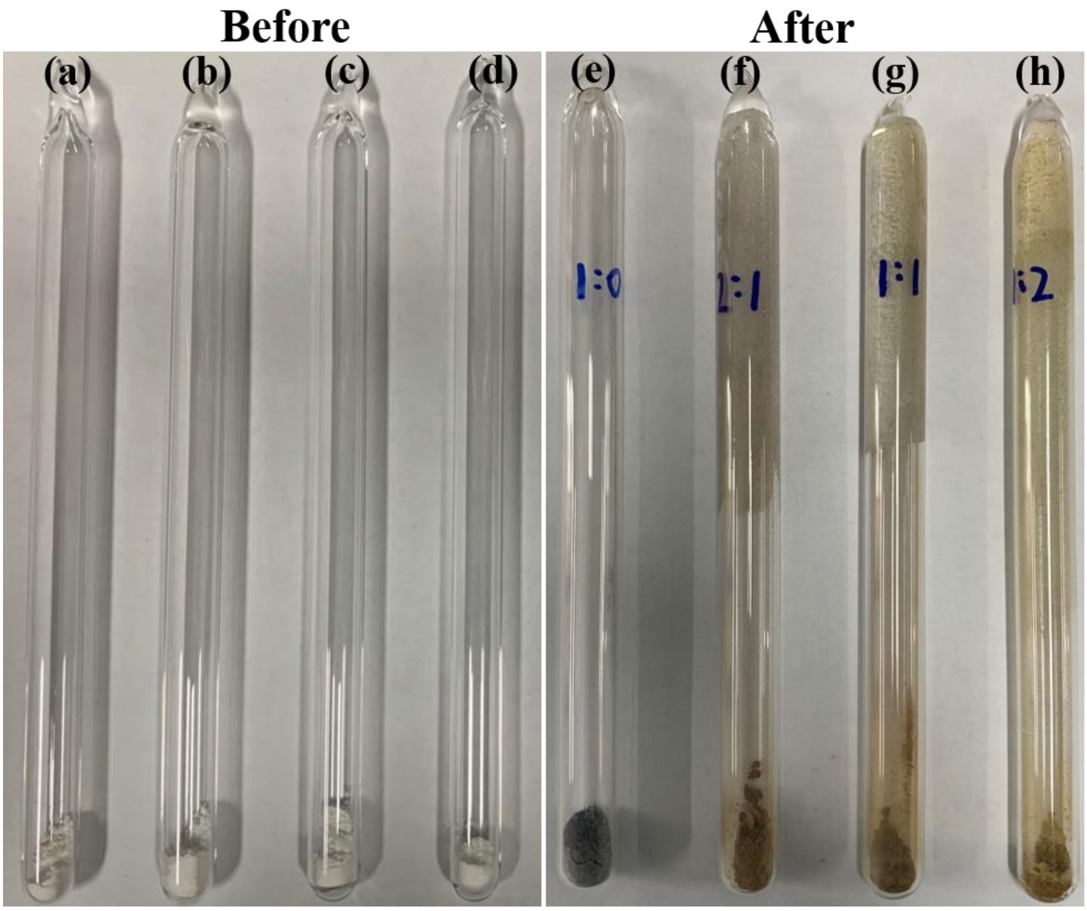

Figure S1. Digital photographs of TiO_2 and urea (a-d) before and (e-h) after the annealing treatment (500 °C, 2h). The weight ratios of TiO_2 to urea are (a, e) 1:0, (b, f) 2:1, (c, g) 1:1 and (d, h) 1:2, respectively.

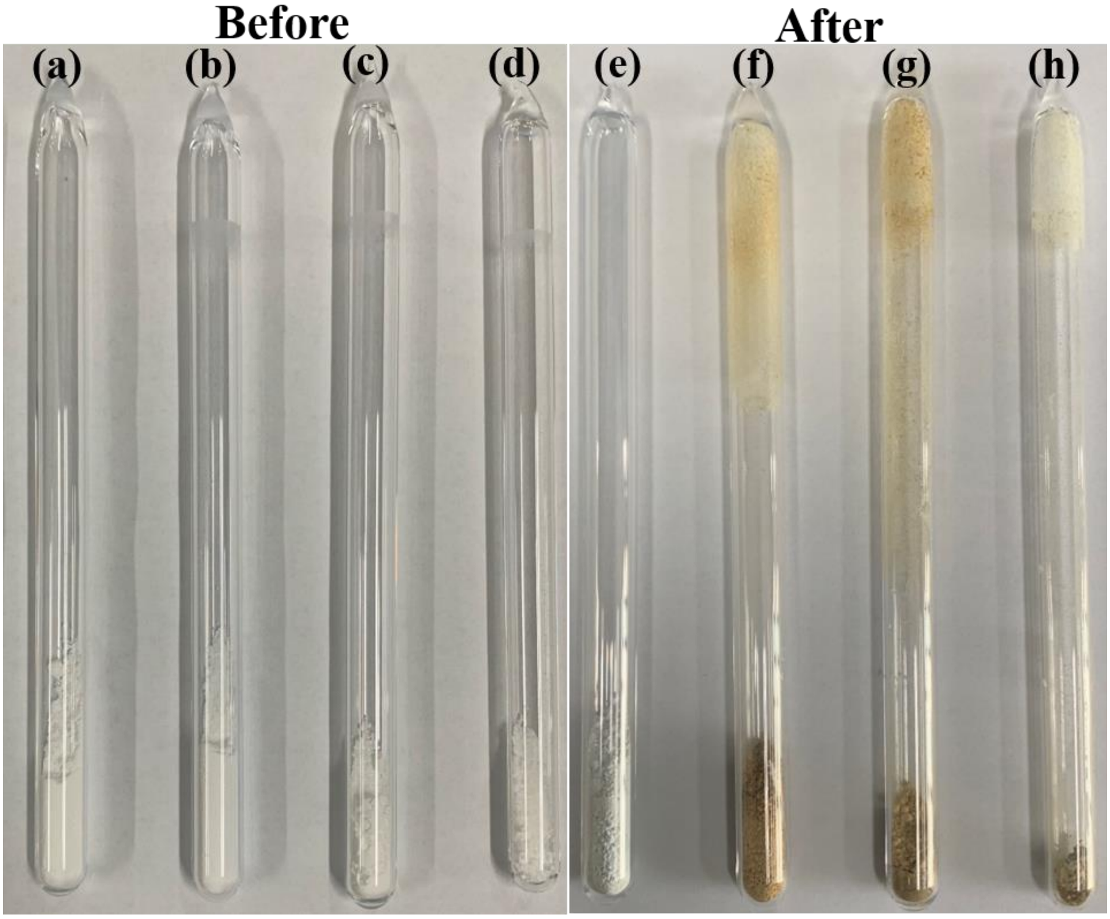

Figure S2. Digital photographs of P25 and urea (a-d) before and (e-h) after the annealing treatment (500 °C, 2h). The weight ratios of P25 to urea are (a, e) 1:0, (b, f) 2:1, (c, g) 1:1 and (d, h) 1:2, respectively.

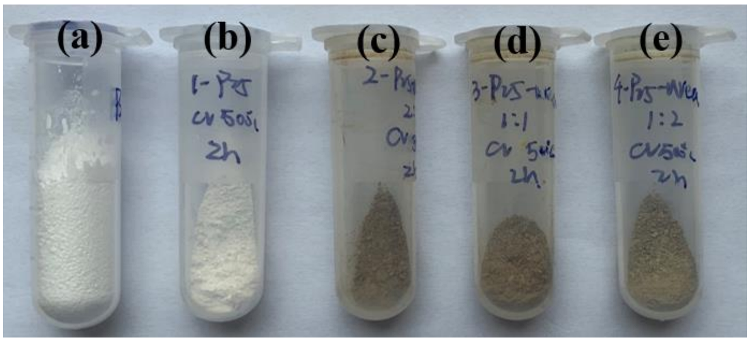

Figure S3. Digital photographs of (a) P25, (b) A-P25, (c) N-P25 (2:1), (d) N-P25 (1:1), and (e) N-P25 (1:2).

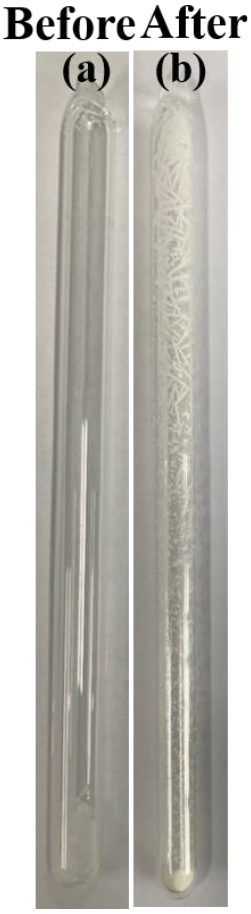

Figure S4. Digital photographs of urea (a) before and (b) after the annealing treatment (500 °C, 2h).

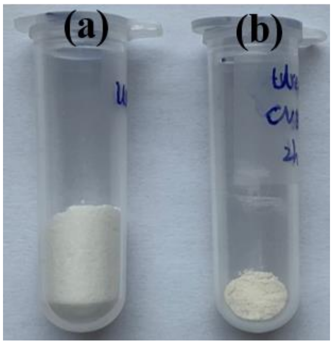

Figure S5. Digital photographs of the urea (a) before and (b) after the annealing treatment (500 °C, 2h).

Table S1. BET surface area of P25, A-P25, N-P25 (2:1), N-P25 (1:1), and N-P25 (1:2).

Sample	BET (m^2/g)
**P25**	47.53
**A-P25**	31.85
**N-P25 (2:1)**	47.54
**N-P25 (1:1)**	48.74
**N-P25 (1:2)**	38.86

Table S2. Comparison of photocatalytic activity of N-TiO_2 photocatalyst for degradation of MO.

Catalysts	Conditions	Removal %	Light source	Preparation method	Ref.
**TON-2**	MO (10 mg/L), Catalyst (1 g/L)	95% at 180 min	Xenon lamp with a 420 nm cut-off filter	Solvothermal method	1
**N550**	MO (20 mg/L), Catalyst (1 g/L)	50% at 300 min	LZC-420	Hydrothermal method	2
**NT3**	MO (15 mg/L), Catalyst (0.3 g/L)	66% at 180 min	Xenon lamp with a 400 nm cut-off filter	Solvothermal method	3
**5N-TiO_2**	MO (5 mg/L), Catalyst (/)	89% at 180 min	Metal halide lamp	Incipient wetness impregnation method	4
**NTNTs-350**	MO (10 mg/L), Catalyst (1 g/L)	96% at 240 min	Fluorescent lamps	Hydrothermal	5
**N-TiO_2**	MO (20 mg/L), Catalyst (0.01 g/L)	61% at 90 min	Mercury lamp	Heat treatment	6
**NT5.0**	MO (20 mg/L), Catalyst (0.1 g/L)	10% at 150 min	Xenon lamp with a 400 nm cut-off filter	Hydrothermal	7
**N-TiO_2 (1:2)**	**MO (25 mg/L), Catalyst(1 g/L)**	**70% at 120 min**	**Xenon lamp with a 420 nm cut-off filter**	**Hydrothermal Annealing treatment**	**This work**

References

1. Yang, G.D.; Jiang, Z.; Shi, H.H.; Xiao, T.C.; Yan, Z.F. Preparation of highly visible-light active N-doped TiO_2 photocatalyst. *J. Mater. Chem.* **2010**, *20*, 5301–5309.
2. Wang, J.; Tafen, D.N.; Lewis, J.P.; Hong, Z.L.; Manivannan, A.; Zhi, M.J.; Li, M.; Wu, N.Q. Origin of photocatalytic activity of nitrogen-doped TiO_2 nanobelts. *J. Am. Chem. Soc.* **2009**, *131*, 12290–12297.
3. Gai, L.G.; Duan, X.Q.; Jiang, H.H.; Mei, Q.H.; Zhou, G.W.; Tian, Y.; Liu, H. One-pot synthesis of nitrogen-doped TiO_2 nanorods with anatase/brookite structures and enhanced photocatalytic activity. *CrystEngComm* **2012**, *14*, 7662–7671.
4. Saien, J.; Mesgari, Z. Photocatalytic degradation of methyl orange using hematoporphyrin/N-doped TiO_2 nanohybrids under visible light: Kinetics and energy consumption. *Appl. Organomet. Chem.* **2017**, *31*, e3755.
5. Peng, Y.; Lo, S.; Ou, H.; Lai, S. Microwave-assisted hydrothermal synthesis of N-doped titanate nanotubes for visible-light-responsive photocatalysis. *J. Hazard. Mater.* **2010**, *183*, 754–758.
6. Xiong, Z.H.; Chen, H.Y.; Lu, L.L.; Shan, R.; Zhang, Y.Y.; Yuan, H.R.; Chen, Y. Nitrogen-doped TiO_2/nitrogen-containing biochar composite catalyst as a photocatalytic material for the decontamination of aqueous organic pollutants. *ACS Omega* **2023**, *8*, 791–803.
7. Zhou, X.S.; Peng, F.; Wang, H.J.; Yu, H.; Yang, J. Preparation of nitrogen doped TiO_2 photocatalyst by oxidation of titanium nitride with H_2O_2. *Mater. Res. Bull.* **2011**, *46*, 840–844.
